# A Cluster-then-label Semi-supervised Learning Approach for Pathology Image Classification

**DOI:** 10.1038/s41598-018-24876-0

**Published:** 2018-05-08

**Authors:** Mohammad Peikari, Sherine Salama, Sharon Nofech-Mozes, Anne L. Martel

**Affiliations:** 1 Medical Biophysics, University of Toronto, Toronto, Canada; 2 Laboratory Medicine and Pathobiology, University of Toronto, Toronto, Canada; 3 Physical Sciences, Sunnybrook Research Institute, Toronto, Canada

## Abstract

Completely labeled pathology datasets are often challenging and time-consuming to obtain. Semi-supervised learning (SSL) methods are able to learn from fewer labeled data points with the help of a large number of unlabeled data points. In this paper, we investigated the possibility of using clustering analysis to identify the underlying structure of the data space for SSL. A cluster-then-label method was proposed to identify high-density regions in the data space which were then used to help a supervised SVM in finding the decision boundary. We have compared our method with other supervised and semi-supervised state-of-the-art techniques using two different classification tasks applied to breast pathology datasets. We found that compared with other state-of-the-art supervised and semi-supervised methods, our SSL method is able to improve classification performance when a limited number of labeled data instances are made available. We also showed that it is important to examine the underlying distribution of the data space before applying SSL techniques to ensure semi-supervised learning assumptions are not violated by the data.

## Introduction

**Figure 1:**
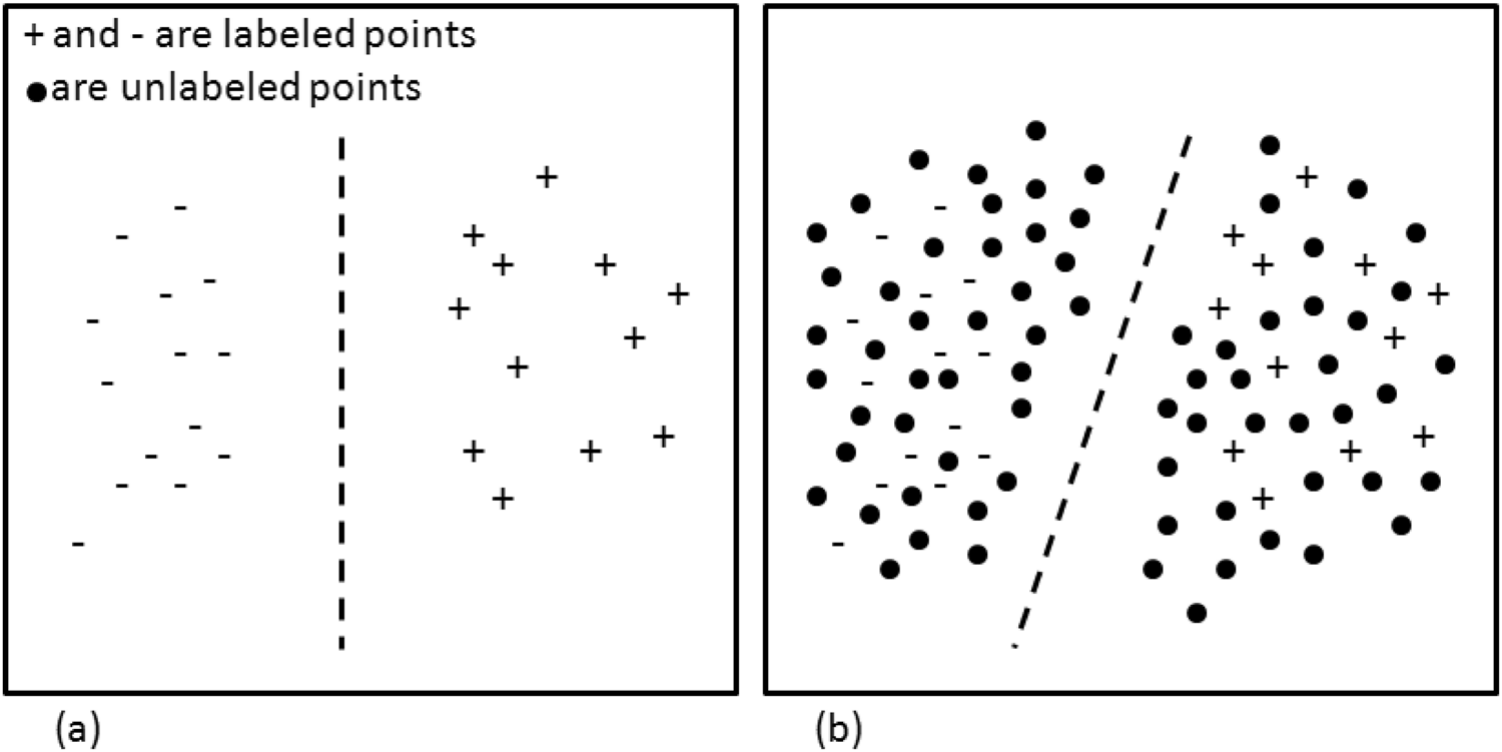
Semi-supervised learning tries to increase the generalization of classification performance by placing the decision boundary through the sparse regions in presence of both labeled and unlabeled data points. (**a**) The decision boundary in presence of labeled data points only, and (**b**) the decision boundary in presence of both labeled and unlabeled data.

Traditionally, there have been two fundamentally different tasks in the spectrum of pattern recognition and machine learning methods. On one side is *supervised learning* in which the goal is to learn a model from labeled data points. The learned model is then applied to an unseen test set and the method is validated based on how successful it was in assigning test data to different classes. The disadvantage of supervised learning techniques is that they are limited to learning from labeled datasets which are often expensive, time consuming, or difficult to generate. If the available labeled dataset is too small and does not represent the true variance of the data space then generalization performance may be poor. This issue is even more decisive in the medical image analysis domain since generating high quality datasets requires the effort of experienced and trained human observers. On the other side of the spectrum are the *unsupervised learning* methods in which unlabeled data points are grouped into clusters that share similar properties. Unlabeled datasets are often easier to acquire and require less human effort to create, however, since the information provided to these techniques is unlabeled, there is no clear way to validate the quality of this approach. In contrast to supervised learning, which only considers labeled data, and unsupervised learning which works only on unlabeled data, semi-supervised learning (SSL) methods work with both labeled and unlabeled data points. Therefore, by using SSL, it is possible to combine the advantages of working with a small labeled dataset to guide the learning process and a larger unlabeled dataset to increase the generalizability of the found solution as shown in Fig. 1^1^.

Pathology images are an important source of diagnostic and prognostic information. With the advent of whole slide scanner technologies, pathology slides are being digitized at microscopic resolution making it possible to store and analyze digital pathology images using computer systems. This has led to a rapidly growing field of research into machine learning techniques that can be used to classify images and provide quantitative information. A major difficulty facing researchers is the availability of labeled training data. Whole slide pathology images are orders of magnitude larger than other medical images and they are more complex. Pathologists use a combination of color, texture and morphological information that varies across multiple scales to interpret images and spend many years learning how to cope with enormous variability in the appearance of specific tissue and disease types. This means that it requires an expert to provide ground-truth labels and it also means that for every new application, additional training and validation data is needed; this makes the use of semi-supervised learning particularly relevant for digital pathology.

In this paper, we present a semi-supervised learning method that analyzes groups of labeled and unlabeled points in multidimensional feature space in order to identify areas of high density and then guides the learning method to place decision boundaries through the regions with low density. We apply this technique to the analysis of digital pathology images of breast cancer.

## Related Works

Semi-supervised learning methods are not commonly used in the pathology image analysis field although they have previously been employed in some applications of medical image analysis to improve classification performances on partially labeled datasets^2–5^. In order to make it possible for semi-supervised learning methods to make the most of the labeled and unlabeled data, some assumptions are made for the underlying structure of data space^1^. Among the assumptions, *smoothness* and *cluster assumption* are the basis for most of the state-of-the-art techniques^6^. In the smoothness assumption, it is assumed that points that are located close to each other in data space are more likely to share the same label, and in the cluster assumption, it is assumed that the data points that belong to one class are more likely to form and share a group/cluster of points. Therefore, the core objective of these two assumptions is to ensure that the found decision boundary lies in low density rather than high density regions within data space.

The most basic and easiest SSL method to apply is self-training^7–10^, which involves repeatedly training and retraining a statistical model. First, labeled data is used to train an initial model and then this model is applied to the unlabeled data. The unlabeled points for which the model is most confident in assigning labels to, are then added to the pool of labeled points and a new model is trained. This process is repeated until some convergence criterion is met. Another family of methods is based on generative models^11–13^, in which some assumptions are made about the underlying probability distribution of data in feature space. The parameters defining the assumed generative model are then found by fitting the model to the data. Graph-based SSL techniques^14–17^, attempt to generate an undirected graph on the training data in which every point on the graph is connected by a weighted edge. The weights are assigned to the edges in such a way that closer data points tend to have larger weights and hence they likely share same labels. Labels are assigned to the unlabeled points by propagating labels of labeled points to unlabeled ones through the edges of the graph with the amount dependent on the edge weights. This way unlabeled points can all be labeled even if they are not directly connected to the labeled points.

The support vector machine (SVM) classifier is an efficient and reliable learning method and to date is one of the best classifiers in terms of performance^18^ over a wide range of tasks. Semi-supervised SVM techniques expand the idea of traditional SVM to incorporate the ability to use partially labeled datasets to learn reliable models while maintaining accuracy. The idea is to minimize an objective function by examining all possible label combinations of the unlabeled data iteratively in order to find low-density regions in the data space to place the decision boundary through^19–22^. Many implementations of the objective functions have been reported in the literature however these are often time inefficient. The reader is referred to Chapelle *et al*.’s work^23^ for a review comparing different methods. Kernel tricks which implement the cluster assumption in SSL have also been proposed^24,25^.

Recently, there have been some attempts to replace the lengthy objective function optimization process of semi-supervised SVMs by cluster analysis^6,26,27^. The concept behind these cluster-then-label techniques for semi-supervised learning^28^ is to first find point clusters of high density regions in data space and then assign labels to the identified clusters. A supervised learner is then used to find the separating decision boundary that passes through low density regions in data space (i.e. between the clusters). In this study, we propose a novel cluster-then-label semi-supervised learning method and compare its performance with other state-of-the-art techniques for two digital pathology tasks; triaging clinically relevant regions of breast whole mount images^29^ and the classification of nuclei figures into lymphocyte, normal epithelial and malignant epithelial objects.

## Methodology

### Proposed Method

In an earlier work^30^, we demonstrated that a semi-supervised cluster-then-label method was able to produce a reliable classification model from small amounts of labeled data. In this study, we propose an improvement of the method proposed in our earlier study^30^ and we carry out an extensive experimental comparison with other state-of-the-art semi-supervised techniques on two different pathology image classification tasks.

#### Clustering Analysis for Semi-supervised learning

Inspired by the work published by Ankerst *et al*.^31^, we propose a cluster-then-label based SSL method that works by finding the underlying structure of points (clusters of points forming high density regions) in the data space. A standard supervised SVM is then employed to find the decision boundary using knowledge about the underlying structure of the data space provided by the clustering analysis. In the Ankerst *et al*.’s^31^ study, an ordering of points in the data space was found based on how points are spatially located around each other. Therefore, spatially closest points become neighbors in the ordering set. The clustering approach presented by Ankerst *et al*.^31^ is unsupervised and finding the clusters from the ordering set is a challenge.

In this paper, our approach in finding spatially closest points in the data space is somewhat similar to the one proposed by Ankerst *et al*.^31^, in the sense that points are grouped in such a way that they form clusters of densely populated points separated by regions with sparsely located points (low density). We consider a semi-supervised seeded approach to finding spatially closest points and checking how inclined unlabeled points are toward each of their surrounding labeled points. The algorithm starts by calculating the *core* radii of the labeled points with respect to all points in the data space. A labeled/unlabeled point *q* is located at a core radius to a labeled point *p* if it is within a circle/sphere with *p* as its center and *ε* as its radius. The value of *ε* for every point *p* is defined as the distance from *p* to the *k*th closest point located within the neighborhood of *p*. Parameter *k* is the minimum number of points located at the neighboring of *p* that could form a cluster and is specified by the user. Thus, the core radius is low in high density regions and high in low density regions. For this study, the value of *k* was set to one tenth of the number of points in the data space. This value of *k* showed a more consistent performance in our preliminary experiments^30^.

The core radii of all labeled points with respect to the whole data space are calculated. We define a distance matrix *D* of the size *l* × *u* where *l* is the number of labeled points and *u* is the number of unlabeled points. The Euclidean distance between the labeled point *p*_*i*_ and the unlabeled point *q*_*j*_ is compared to the core distance *ε*_*i*_, and *d*_*ij*_ is defined as the maximum of these two values. Therefore, this could be written as:

1 $${d}_{ij}=max({\varepsilon }_{i},||{p}_{i}-{q}_{j}{||}_{2});{\forall }_{i=1}^{l}\,{\rm{and}}\,{\forall }_{j=1}^{u}$$

where *ε*_*i*_ is the core radius of the labeled point *p*_*i*_, *l* is the number of labeled points, and *u* is the number of unlabeled points.

**Figure 2:**
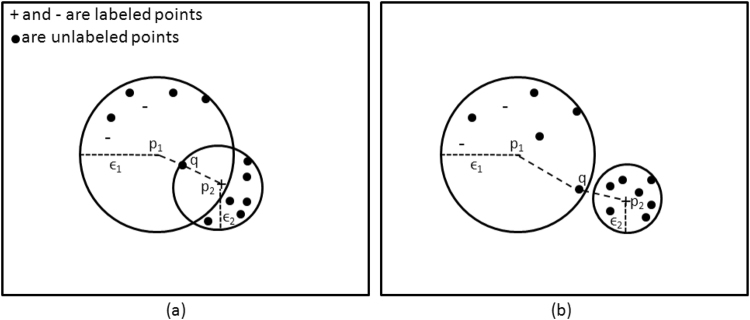
Example showing different scenarios where an unlabeled point *q* is located with respect to the clusters formed by labeled points *p*_1_ and *p*_2_. (**a**) An unlabeled point *q* is within the core radii of two labeled points *p*_1_, and *p*_2_. Since *ε*_2_ < *ε*_1_, *q* is assigned to *p*_2_ despite the fact that, according to the Euclidean distance, it is actually closer to *p*_1_. (**b**) An unlabeled point *q* is within the core radius of the labeled point *p*_1_ but not *p*_2_. Since *ε*_1_ > ||*p*_2_ − *q*||_2_, *q* is again assigned to *p*_2_. *ε*_1_, and *ε*_2_ are the core radii of the labeled points *p*_1_ and *p*_2_ respectively. Please note that the *ε*_1_ and *ε*_2_ are different based on the density of points surrounding them. In this representation, *k* is set to be 7.

Once the matrix *D* has been populated using expression (1), it is used to find the closest labeled point for each unlabeled point. Figure 2 shows an example of how core radii are useful in assigning unlabeled points to different clusters and highlights differences between conventional clustering methods. In Fig. 2(a) the unlabeled point *q* is located within the core radii of both the labeled points *p*_1_ and *p*_2_. Since *ε*_2_ < *ε*_1_, *q* is assigned to *p*_2_ despite the fact that, according to the Euclidean distance, it is actually closer to *p*_1_. In Fig. 2(b) the unlabeled point *q* is within the core radius of *p*_1_ and lies outside of the core radius of *p*_2_ however, since *ε*_1_ > ||*p*_2_ − *q*||_2_, *q* is again assigned to *p*_2_. In cases where an unlabeled point *q* is equidistant between two points with different labels, *q* is assigned a negative label. This, however, rarely happens in practice as distances are represented as floating point values. Hence, this method finds and groups the points that match both the smoothness and cluster assumptions in SSL and is referred to as *Semi-supervised Seeded Density-based* (S^3^DB) clustering hereafter.

After the groups of points that form clusters are identified and the underlying structure of the data space is learned (using S^3^DB), this knowledge is given to an SVM classifier with radial basis function (RBF) as kernel to find the maximum margin boundary that passes through the sparse regions.

### Datasets

#### Pathology Triaging Image Dataset

Recently^29^, we addressed the problem of triaging digital pathology images and employed a supervised learning method to distinguish between different relevant or irrelevant breast tissue regions. Here, we consider a more extensive dataset and focus on the statistical learning aspect of the problem. The goal is to achieve a high sensitivity of at least 95% in detecting relevant regions while maintaining the highest possible specificity.

**Figure 3:**
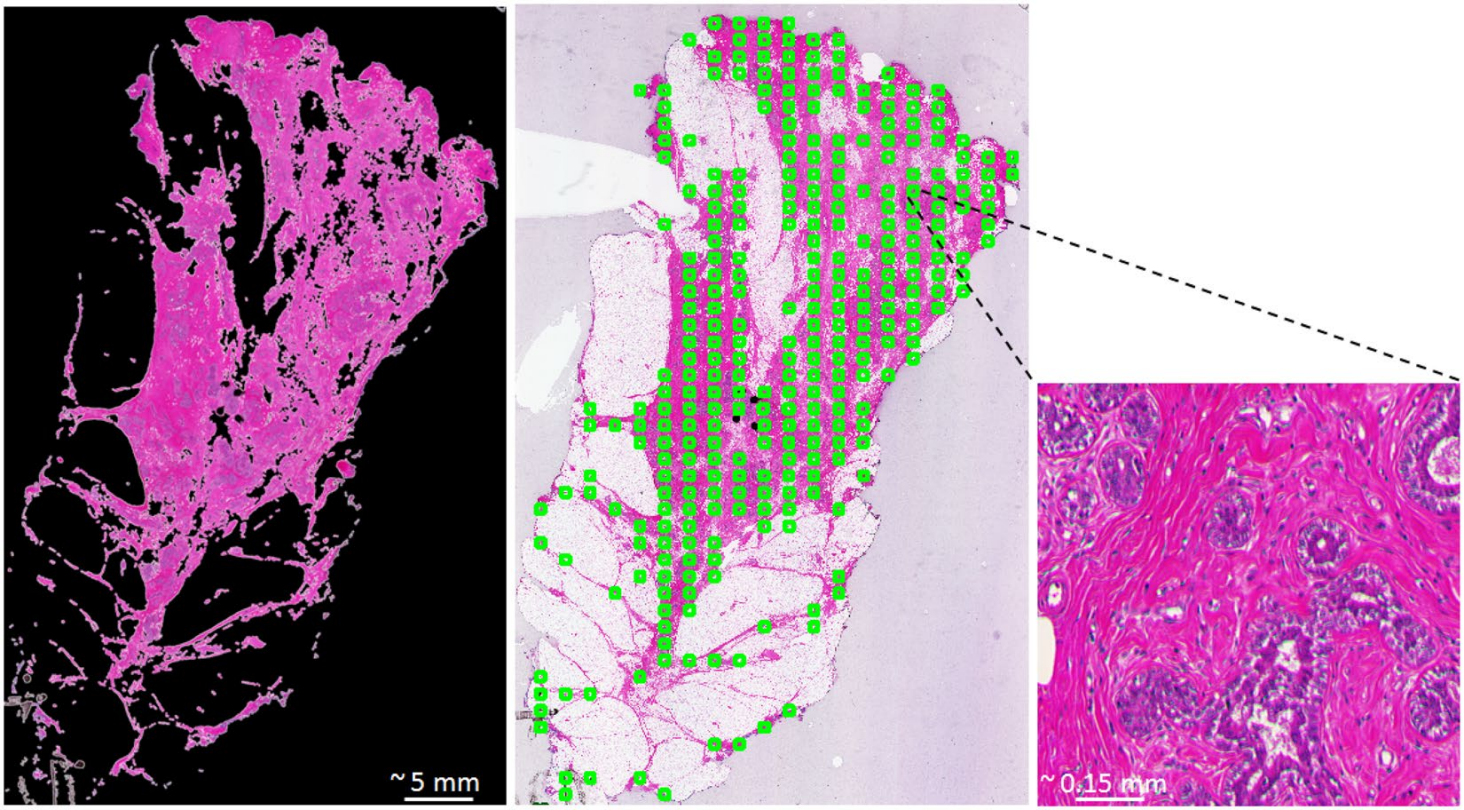
Adaptive thresholding and morphological operations were applied to remove clearly irrelevant structures before patch selection for the data collection process (left), 512 × 512 pixel uniformly spaced box patches (in green) on tissue regions (middle), and an example of a 512 × 512 pixel image patch picked from the WMI (right).

Data Collection: To generate a ground-truth dataset, we have used whole-mount images (WMIs)^32^ of 30 breast lumpectomy specimens stained with hematoxylin and eosin (H&E) (*n* =  150 WMIs). The slides corresponding to 28 of the patients were scanned at 5X magnification (135 WMIs, 2 *μ*m/pixel) and 2 of them were scanned at 10X (15 WMIs, 1 *μ*m/pixel). Patches of 512 × 512 pixels (1 mm^2^ for 5X and 0.25 mm^2^ for 10X images) were cropped from each WMI at the highest magnification by overlaying a grid of uniformly spaced squares on the previously preprocessed (adaptive thresholding and morphological operations) tissue regions (Fig. 3). The collaborating pathologist then labeled patches from the 2 patients scanned at 10X magnification (15 WMIs, 2849 patches) and 8 patients scanned at 5X magnification(115 WMIs, 2302 patches labeled, 2100 patches unlabeled). For each patch, the pathologist evaluated the presence of diagnostically relevant information corresponding to each tissue type. According to the pathologist’s annotations, diagnostically relevant features include cancers, atypias, microcalcifications and lymphovascular invasion, and irrelevant features include fat, stroma, normal ducts and lobules. To assess inter-observer variability when labeling the triaging ground-truth set, a random subset of 1500 patches were evaluated by a second pathologist. The Kappa agreement coefficient between the two pathologists was *κ* = 0.77.

**Figure 4:**
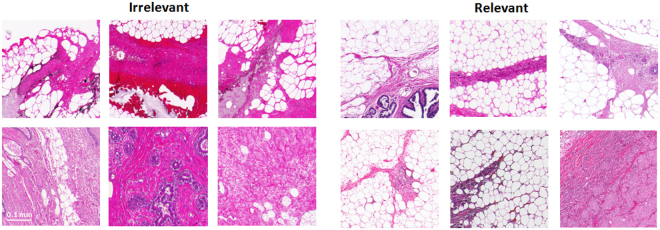
A subset of cropped image patches used as ground-truth in the triaging dataset used for this study with their labels. It is clear that the clinically relevant information may have covered different portions of the patches in the dataset since they were randomly picked from different areas within the tissue region.

Figure 4 shows a subset of this ground-truth set. We have also added 1500 unlabeled image patches from the remaining 20 patients scanned at 5X (20 WMIs). This set of unlabeled patches was used to improve the generalization performance of the learning models as mentioned in section 3.3.1.

Texture Feature Extraction from Patches: To retrieve texture features from image patches, they were converted from RGB to Lab colorspace and the normalized luminance channel was divided into smaller non-overlapping tiles of size 32 × 32 pixels. Root filter set (RFS)^33^ texture filters were used to highlight different textures from image tiles. First order statistical measures (mean, mode, standard deviation, skewness and kurtosis) were calculated from the maximal filter responses along all filter orientations of each scales to combine the texture information. To regroup all extracted information from individual tiles and form one numerical representative per image patch, the bag of words (BoW)^34^ technique was used with a previously found optimum dictionary size of 100^29^. The calculated 100-dimensional histograms of words per individual image patch were used to train and evaluate the statistical learning techniques presented in this paper. We used the RBF kernel of the SVM classifier implemented in libsvm library^35^ to find the best separating hyperplane between the two classes.

#### Nuclei Figure Classification Dataset

Recently^36^, we developed an automated method to assess cancer cellularity in breast tissue removed after neoadjuvant chemotherapy (NAT). As a part of the pipeline, we developed a method to classify nuclei figures into three classes of lymphocyte (L), benign epithelial (B) and malignant epithelial (M) figures from a dataset of image patches annotated by a pathologist. Here, we used the same dataset to validate the proposed SSL technique.

Data Collection: H&E stained sections from 92 post-NAT lumpectomy specimens were scanned at 20X magnification (0.5 *μ*m/pixel). The whole slide images (WSIs) were annotated by an expert pathologist using the Sedeen Viewer^37^ (Pathcore, Toronto, Canada). A total of *n* = 166 rectangular regions of interest (ROIs) were defined on the 92 WSIs and, within these ROIs, the centers of nuclei figures were labeled as either lymphocyte, benign epithelial, or malignant epithelial. Nuclei that were out of focus, out of plane, or could not be categorized, were not marked by the pathologist. More than 30,000 nuclei figures (*n* = 3,868 lymphocyte, *n* = 10,407 benign epithelial, and *n* = 16,419 malignant epithelial figures) were marked from all 116 ROI patches.

Nuclei Feature Extraction from Patches: In order to train the proposed SSL method, the nuclei have to be segmented first. We have developed a segmentation method^38^ that works by manipulating the original RGB colorspace of the image patches to better identify foreground nuclei figures. Multilevel thresholding and marker controlled watershed algorithms were then used to extract nuclei regions and divide overlapping nuclei figures. The nuclei segmentation method achieved an F1-score of 0.9 when tested against a publicly available dataset of 7931 nuclei from 36 images^39^. The effect of color variation from the image patches was reduced by standardizing their color to a reference image as explained in our recent study^36^. The segmentation method was able to segment more than 72% of the ground-truth nuclei figures (*n* = 21,779) from the 166 ROI patches. The segmented figures were used to extract 125-dimensional feature vectors from individual nuclei figures based on intensity, morphological, textural, and spatial properties describing their differences among the three classes^36^. Table 1 summarizes the datasets used to validate and compare performances of the supervised and semi-supervised learning methods described in section 3.1.

**Table 1:** Summary of the data proportions used in each validation stage for the two pathology datasets used in this study.

	Dataset	
	Triaging Image Dataset	Nuclei Figure Classification
# of labeled instances in training set	2,302	13,821
# of unlabeled instances in training set	2,100 + 1,500	49,000
# of instances in test set	2,849	7,958
Dimensionality of feature vectors	100	125

## Experimental Setup

### Comparison with State-of-the-art Methods

We compare our proposed SSL method (S^3^DB + SVM) with a range of successful supervised and semi-supervised methods in the literature.

#### Method for supervised learning

The standard supervised SVM technique implemented in the libsvm^35^ library was considered to find the separating decision hyperplane. Here we used the RBF kernel and a similar parameter optimization approach to other methods described in this paper was followed, as explained in the subsequent section. Let *X* = *x*_1_, *x*_2_, …, *x*_*l*_ be the set of d-dimensional labeled points with *Y* = *y*_1_, *y*_2_, …, *y*_*l*_ be their labels. The SVM technique works by minimizing the optimization function presented in equation (2) to find the maximum margin hyperplane parameters dividing the two classes.

2 $$mi{n}_{\overrightarrow{w},b,\xi ,C}\frac{1}{2}||\overrightarrow{w}{||}^{2}+C\sum _{i=1}^{l}\,{\xi }_{i}$$

subject to:

$$\begin{array}{c}{{\rm{\forall }}}_{i=1}^{l}\,:{y}_{i}[\overrightarrow{w}.{\rm{\Phi }}(\overrightarrow{{x}_{i}})+b]\ge 1-{\xi }_{i}\\ \,\,\,\,{{\rm{\forall }}}_{i=1}^{l}\,:{\xi }_{i} > 0\end{array}$$

where $\overrightarrow{w}$ and *b* are the parameters defining the maximum margin hyperplane, Φ (.) is the kernel function, *C* is the parameter defining the trade-off between the margin size and misclassified examples, and *ξ* is the slack variable.

#### Methods for semi-supervised learning

*a*) *semi-supervised Fuzzy c-mean* (*ssFCM*) *clustering* + *SVM*: this method has been previously employed for semi-supervised learning^2,27,40,41^. The idea is to first apply the semi-supervised clustering to both labeled and unlabeled data to find the underlying structure of the space (hard labeling) and then a supervised classifier is trained on the labeled data. The semi-supervised version of the original unsupervised FCM in particular is useful to provide a prior knowledge on the structure of the space in the form of labels^27^. Therefore, in the following optimization problem, the first term is to discover the data space structure of the labeled data and the second term takes care of the unlabeled data. Let *X* = *x*_1_, *x*_2_, …, *x*_*l*_ be the set of d-dimensional labeled points with *Y* = *y*_1_, *y*_2_, …, *y*_*l*_ be their labels and ${X}^{\ast }={x}_{1}^{\ast },{x}_{2}^{\ast },\ldots ,{x}_{u}^{\ast }$ be the set of unlabeled points, then the ssFCM objective function can be written as:

3 $$min{\rho }_{m}=\sum _{i=1}^{c}\sum _{j=1}^{l}\,{u}_{ij}^{m}||{X}_{j}-{V}_{j}{||}_{2}+\sum _{i=1}^{c}\sum _{j=1}^{u}\,{u}_{ij}^{\ast m}||{X}_{j}^{\ast }-{V}_{j}{||}_{2}$$

where *c* is the number of classes (*c* = 2 for binary classification), *m* is the degree of fuzziness (we set *m* = 2), *V* represents the set of prototypes corresponding with each class, *U* and U^*^ are matrices that define the fuzzy membership values for the labeled and unlabeled data points respectively and *u*_*ij*_ is the probability that the *j*th labeled data point belongs to class *i*. The maximum number of iterations for the experiments using this method was set to 1000 rounds.

We have used the RBF-SVM classifier in conjunction with the semi-supervised FCM method similar to the one presented by Gan *et al*.^27^. The parameters of the SVM classifier was optimized using a similar strategy to other methods described in this paper as explained in section 3.3.

*b*) *TSVM*^19,42^: this SSL method is one of the most successful implementations of the semi-supervised SVM technique in terms of performance^23^. The algorithm starts by learning a partially complete model using the labeled data only and then applies this to the unlabeled data. The method then improves the initial solution by switching the labels assigned to the unlabeled data to decrease the objective function after each iteration. The label switching mechanism is important to ensure the balancing constraints between the two classes are maintained. Therefore, the main objective function to be minimized in this method is as follow:

4 $$mi{n}_{{y}_{1}^{\ast },\ldots ,{y}_{u}^{\ast },\overrightarrow{w},b,\xi ,{\xi }^{\ast },C,{C}^{\ast }}\frac{1}{2}||\overrightarrow{w}{||}^{2}+C\sum _{i=1}^{l}\,{\xi }_{i}+{C}^{\ast }\sum _{j=1}^{u}\,{\xi }_{j}^{\ast }$$

subject to:

$$\begin{array}{c}{\forall }_{i=1}^{l}\,:{y}_{i}[\overrightarrow{w}.{\rm{\Phi }}(\overrightarrow{{x}_{i}})+b]\ge 1-{\xi }_{i}\\ {\forall }_{j=1}^{u}\,:{y}_{j}^{\ast }[\overrightarrow{w}.{\rm{\Phi }}(\overrightarrow{{x}_{j}^{\ast }})+b]\ge 1-{\xi }_{j}^{\ast }\\ {\forall }_{i=1}^{l}\,:{\xi }_{i} > 0\\ {\forall }_{j=1}^{u}\,:{\xi }_{j}^{\ast } > 0\end{array}$$

### Visualization and Cluster Separability

To visualize the underlying distributions of data spaces used in this study in lower dimensions, t-distributed Stochastic Neighbor Embedding (t-SNE) was used^43^. It is an iterative method which maps data points into lower dimensional space in such a way that the distances between points correspond to their similarity. Also, we have used Fisher Discriminant Ratio (FDR)^44–46^, as a measure of cluster separability. FDR measures the cluster separability by calculating the square of the difference between means of points in each cluster divided by the sum of square of their standard deviations:

5 $$FDR=\frac{{({\overline{m}}_{1}-{\overline{m}}_{2})}^{2}}{{\overline{s}}_{1}^{2}+{\overline{s}}_{2}^{2}}$$

where ${\overline{m}}_{1}$ and ${\overline{m}}_{2}$ are the mean of points; and ${\overline{s}}_{1}$ and ${\overline{s}}_{2}$ are the standard deviations of points in clusters 1 and 2 respectively.

### Experimental Design

In order to evaluate the performance of the proposed SSL technique and compare with state-of-the-art methods, the following validation steps were taken.

#### Triaging Image Dataset

Here, the dataset described in section 2.2.1 was divided into a training set containing the patches scanned at 5X magnification, and a testing set containing the 2 patient datasets scanned at 10X (*n* = 2849 labeled image patches). The training data was further subdivided into two components; one part contained the labeled and unlabeled patches from the 8 patients reviewed by the pathologist (*n* = 4402 patches, 307 relevant, 1995 irrelevant, and 2100 unlabeled image patches with the mean and standard deviation of 283 ± 90 labeled patches and 267 ± 243 unlabeled patches per patient) and the second part consisted of the of 1500 unlabeled image patches taken from the remaining patients’ WMIs (section 2.2.1).

An 8-fold patient-wise cross-validation scheme was used to train and validate the performance of learning methods. The optimum SVM-RBF parameters were chosen by examining a range of possible SVM trade-off parameter (*C*) and the kernel width (*γ*) values on the training set. The additional set of 1500 unlabeled image patches (section 2.2.1) was included in all folds of the cross-validation scheme.

Validation step for semi-supervised methods: For every fold in semi-supervised learning methods, one or more of the patient datasets were randomly selected to be the labeled set (unlabeled images of chosen patients were kept unlabeled) and labels of the rest of patients were kept hidden (unlabeled set).

Validation step for supervised method: Similarly, for the supervised learning method, in every fold one or more patient datasets were randomly selected to be the labeled set (unlabeled images of chosen patients were discarded) and rest of the patients data were also discarded.

The randomly selected patients, dictionary of words, and histograms of words were kept the same to form paired labeled sets in every fold of each experiment. To do a fair comparison between different methods, we defined the optimum SVM-RBF parameter set by first identifying all sets that produced a sensitivity of 95%; from which the set with maximum specificity was chosen.

Validation using an unseen set: To compare the generalization performance of the methods, the median of the optimized parameters found in all 8-folds of the cross-validation was considered to train an overall model using all training images. For semi-supervised methods, one or more of patients were randomly chosen to form the labeled set and the labels of the rest of patients were kept hidden. For the supervised method also, one or more of patients data were chosen to form the labeled set and the rest of patients data were discarded. The overall performance of the trained model was assessed using the the two unseen patient cases in the test set. To match our previously trained models on 5X magnified images, the test image patches, which were scanned at 10X magnification, were down-sampled.

#### Nuclei Figure Classification Dataset

The aim of this experiment was to see whether adding many unlabeled instances to an already large set of labeled instances improved the classification performance when comparing an SSL technique with a supervised learning method. A cascaded learning approach was used to first train a classifier to distinguish between lymphocyte versus epithelial figures (L vs. BM) and then to distinguish between benign versus malignant classes (B vs. M).

The supervised SVM was trained using *n* = 13,821 labeled nuclei figures (*n* = 2,260 Lymphocytes, *n* = 3,157 Benign epithelial, and *n* = 8404 Malignant epithelial figures). Both the labeled figures and an additional *n* = 49,000 unlabeled figures were used by the semi-supervised methods.

For both supervised and semi-supervised training, a 5-fold cross-validation was performed to assess the performance of the learning methods. In this experiment, the best parameters were chosen in such a way as to maximize the accuracy.

Once the best parameters had been selected a final model was trained on the whole training dataset using the median of the best parameters in all 5 folds and this was applied to an unseen test set of *n* = 7,958 nuclei figures to evaluate the generalizability of the trained models.

## Results

### Comparing Classification Performances

**Figure 5:**
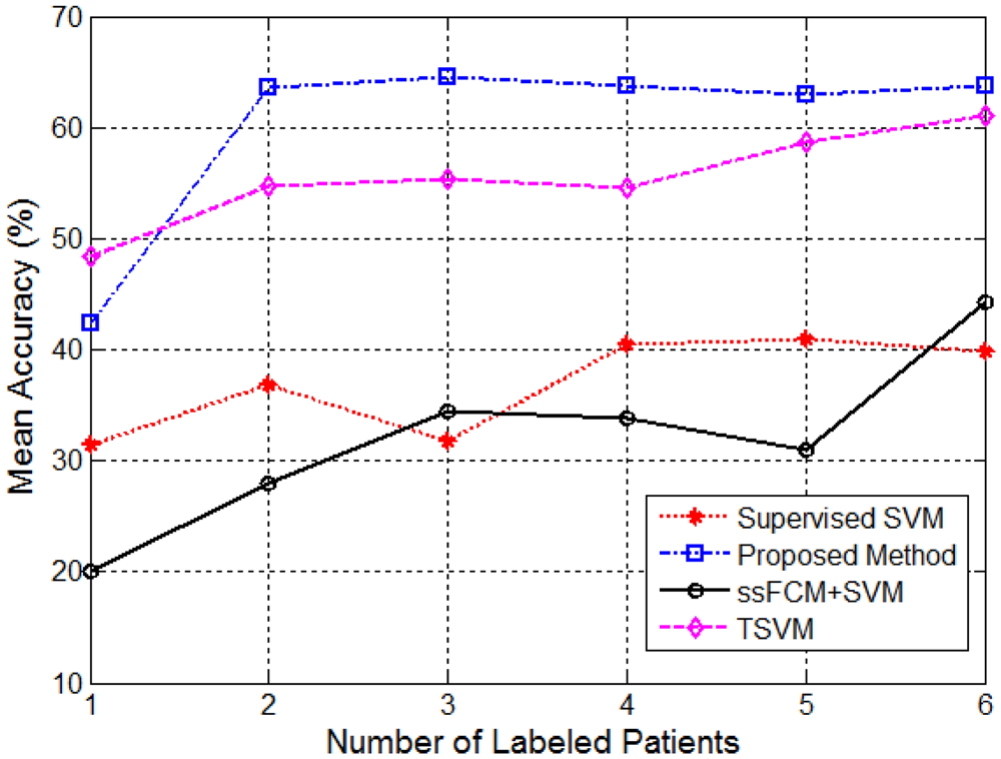
Comparison of mean classification accuracy for 8-fold subject-wise cross-validation of the four supervised and semi-supervised methods discussed in this paper for the breast pathology dataset at different labeled portions. To have a fair comparison, results are reported at an operating point of 95% sensitivity.

Mean accuracy of the subject-wise cross-validated experiments are shown in Fig. 5 for different number of patients chosen to be the labeled set from the pathology triaging image dataset. As can be seen from Fig. 5, our clustering-based SSL technique (S^3^DB + SVM) achieved a superior performance compared to the other state-of-the-art supervised and semi-supervised methods.

Table 2 summarizes the cross-validated performance of different methods on triaging image dataset at an operating point of 95% sensitivity. A pairwise Wilcoxon Signed-Rank test using 8 cross-validated accuracy values was used to compare each method at a given number of labeled patients to that of our proposed SSL technique. For each method tested, we had 6 comparisons, therefore for a two-tailed test with a 5% type I error the Bonferroni adjusted *α*-value = 0.004. Although none of the comparisons achieved statistical significance against this adjusted threshold, there is an increasing trend in the classification performance of our proposed method compared to other techniques. It is clear by looking at the specificity column that in most cases our method maintained higher specificity values at 95% sensitivity compared to the other methods at all individual experiments (except when number of labeled patients = 1). It is also interesting to note that the average train time of our method is significantly lower than the TSVM technique, which requires a heavy optimization on a 64-bit Intel(R) Xeon(R) CPU (at 3.50 GHz) machine. The supervised SVM method however, took the least amount of time to train a model using the labeled data made available to it. Table 3 summarizes the performance of the four methods using an overall model trained in the first validation phase using number of labeled patients as 3 on a totally unseen test set. The test set consisted of two patient cases scanned at 10X magnification. It is clear that our method consistently performs better in classifying image patches compared to the other supervised and semi-supervised techniques in a totally unseen test set.

**Table 2:** Results comparing the mean performance of the 8-fold subject-wise cross-validated methods on triaging image dataset. Results are reported at an operating point of 95% sensitivity. A pairwise Wilcoxon Signed-Rank test was used to check for statistical significance in accuracy performances of each method compared with our proposed method. No statistically significant difference was observed between the pairs performances after adjusting for multiple testing using the Bonferroni method (adjusted *α*-value = 0.004).

Method	# of Labeled Patients	AUC	Accuracy (SD) (%)	95% CI	Specificity (%)	p-value	Train Time (min)
SVM	1	0.71	31.4 (17.2)	[17.0, 45.8]	19.9	0.253	1 ± 0.1
	2	0.76	36.8 (23.8)	[16.9, 56.7]	27.8	0.015	2 ± 0.2
	3	0.75	31.8 (18.3)	[16.5, 47.1]	21.4	0.007	4 ± 0.2
	4	0.76	40.4 (22.6)	[21.5, 59.3]	32.4	0.007	6 ± 0.5
	5	0.78	41.0 (24.1)	[20.8, 61.1]	33.6	0.039	8 ± 0.8
	6	0.79	39.8 (23.6)	[20.1, 59.5]	31.9	0.007	13 ± 1
ssFCM + SVM	1	0.62	20.1 (15.7)	[7.0, 33.2]	5.2	0.007	97 ± 11
	2	0.70	28.0 (28.2)	[4.4, 51.6]	18.1	0.007	120 ± 13
	3	0.72	34.4 (27.4)	[11.5, 57.3]	25.3	0.007	112 ± 16
	4	0.73	33.9 (26.5)	[11.7, 56.6]	24.1	0.007	126 ± 16
	5	0.74	31.0 (27.1)	[8.3, 53.6]	22.5	0.007	131 ± 13
	6	0.78	44.3 (21.7)	[26.1, 62.4]	34.2	0.039	138 ± 17
TSVM	1	0.81	48.4 (18.3)	[33.1, 63.7]	39.2	0.583	6178 ± 3408
	2	0.83	54.7 (17.8)	[39.8, 69.6]	46.4	0.546	7100 ± 3663
	3	0.83	55.4 (18.3)	[40.1, 70.7]	46.8	0.541	8008 ± 4238
	4	0.83	54.6 (15.9)	[41.3, 67.9]	47.8	0.148	7093 ± 4113
	5	0.84	58.7 (17.5)	[44.1, 73.3]	52.8	0.296	7432 ± 4259
	6	0.85	61.0 (16.1)	[47.5, 74.5]	54.4	0.541	6551 ± 3536
S^3^DB + SVM	1	0.69	42.3 (16.9)	[28.2, 56.4]	33.1	—	105 ± 21
	2	0.83	63.6 (18.4)	[48.2, 79.0]	59.1	—	88 ± 11
	3	0.84	64.5 (19.9)	[47.9, 81.1]	60.2	—	114 ± 11
	4	0.84	63.8 (17.8)	[48.9, 78.7]	59.1	—	85 ± 11
	5	0.84	62.9 (17.2)	[48.5, 77.3]	57.7	—	137 ± 18
	6	0.84	63.7 (18.1)	[48.6, 78.8]	59.6	—	81 ± 9

**Table 3:** Results comparing the performance of the four methods using an overall trained model from triaging image dataset using 3 patients data only as labeled set on a totally unseen test set of 2 patient scanned at 10X magnification.

Method	AUC	Accuracy (%)	Sensitivity (%)	Specificity (%)
Supervised SVM	0.81	42	93	38
ssFCM + SVM	0.59	29	91	24
TSVM	0.85	49	94	45
S^3^DB + SVM	0.86	53	94	49

Table 4 summarizes the mean performance of the 5 fold cross-validation on *n* = 13,821 labeled nuclei figures combined with *n* = 49,000 unlabeled objects using our proposed SSL technique compared with supervised SVM method trained on the labeled portion only. No statistically significant difference in performance was observed between the accuracy pairs of the supervised SVM and our proposed SSL method in Table 4 using a pairwise Wilcoxon Signed-Rank test.

**Table 4:** Mean performance of 5-fold cross-validation on nuclei figure classification dataset using S^3^DB + SVM semi-supervised method (*n* = 13821 labeled nuclei objects and *n* = 49000 unlabeled ones) and supervised SVM method (*n* = 13821 labeled nuclei objects).

Method	Task	AUC	ACC (%)	Sens. (%)	Spec. (%)
Supervised SVM	L vs. BM	0.97	95 (±0.1)	79	99
	B vs. M	0.86	83 (±0.5)	57	92
S^3^DB + SVM	L vs. BM	0.95	93 (±0.4)	74	97
	B vs. M	0.73	74 (±0.5)	7	99

Table 5 summarizes the performance of applying the models generated from both supervised and semi-supervised methods on the training part on an independent testing set of *n* = 7,958 nuclei figures. From Tables 4 and 5 it is clear that the proposed SSL method did not fit well for this dataset and the performance is poor compared to the supervised SVM method.

**Table 5:** Performance of applying the generated model from dataset used in Table 4 on *n* = 7958 nuclei objects from an independent testing set using S^3^DB + SVM and supervised SVM methods.

Method	Class	ACC (%)	Sens. (%)	Spec. (%)
Supervised SVM	L	92	80	94
	B	75	50	92
	M	77	91	63
S^3^DB + SVM	L	87	67	91
	B	60	3	99
	M	60	97	20

### Comparing Cluster Separability Measures

**Figure 6:**
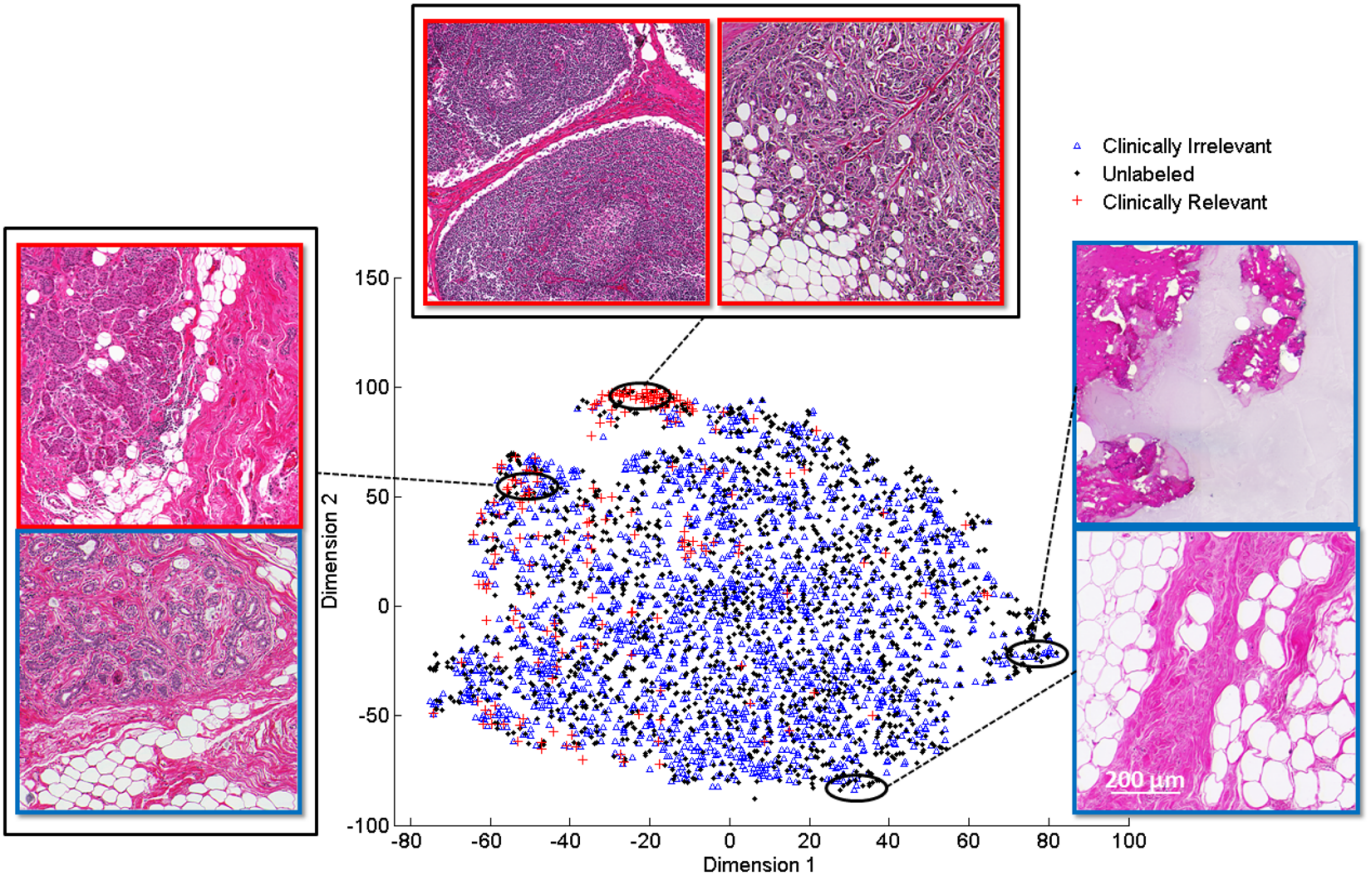
2D visualization of the triaging image dataset feature space using t-SNE^43^. Every point in this plot represents an image patch from the dataset. As can be seen, relevant versus irrelevant images form separable clusters in this visualization.

**Figure 7:**
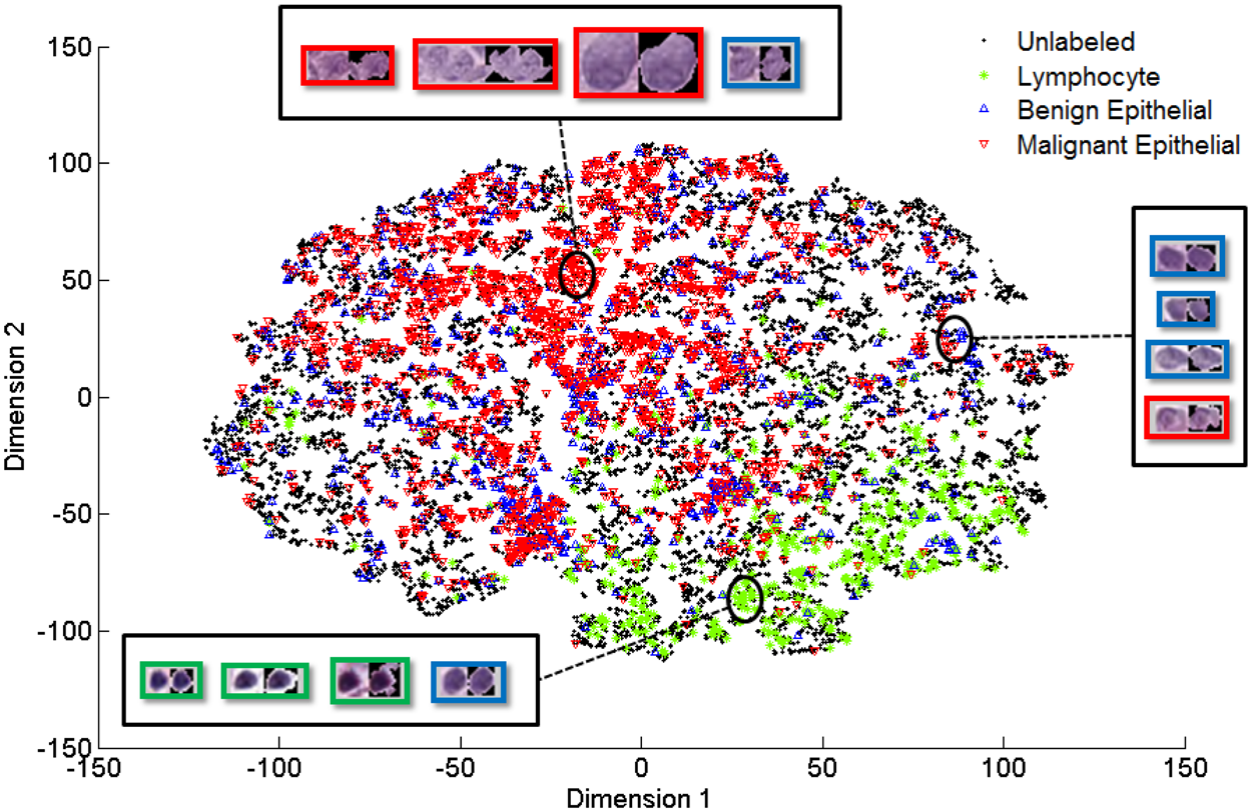
2D visualization of the feature space from nuclei figure classification dataset using t-SNE^43^. Every point in this plot represents a nuclei figure from the dataset. As can be seen, lymphocyte figures versus the other two classes are better separated while benign versus malignant epithelial figures do not form separable clusters.

In order to have a sense of how separable the clusters of each class are with respect to each other, FDR measures are summarized in Table 6. From Table 6 we can see that as the FDR measure increases (relevant vs. Irrelevant and L vs. BM) the classes in each dataset tend to form separable clusters while in case of B vs. M where separable clusters are not formed the FDR measure is low. Furthermore, in order to visually compare the distribution of different class labels in feature spaces of both datasets, their dimensions were reduced using the t-SNE method^43^. Figure 6 shows the dimensionality-reduced data space of the triaging image dataset with every point representing an image patch. Similarly, Fig. 7 shows the data space of the nuclei figure classification dataset with every point representing a nuclei figure. Considering Fig. 6, it can be seen that the relevant and irrelevant classes form separable clusters in the feature space while considering Fig. 7, it can be observed that lymphocyte class is better separated compared to the other two classes. Comparing benign versus malignant epithelial classes in the same figure we see that they do not tend to form separable clusters of points thus violating the cluster assumption of SSL.

**Table 6:** Fisher Discriminant Ratio (FDR) measures for different classes from the datasets used in this study.

Dataset	Classes	Fisher Discriminant Ratio (FDR)
Triaging Image Dataset	Relevant vs. Irrelevant	0.39
Nuclei Figure Classification	L vs. BM	0.27
	B vs M	0.02

There is a slight imbalance between the classes in both triaging image dataset and nuclei figure dataset. To determine whether this affected performance, we repeated the training and testing to check the consistency of the classification performance for both datasets, the weight values of the SVM models for minority class were set to the ratio of the number of data samples in the majority class to that of the minority class. The classification performance was found to be consistent for both the datasets suggesting that the class imbalance did not affect the classification performance.

## Discussion and Conclusion

In this study, we proposed a cluster-then-label based semi-supervised technique to find the underlying structure of the data space and provide this knowledge to train a reliable model. We have compared and validated this technique with other state-of-the-art supervised and semi-supervised methods for triaging breast digital pathology image patches and classifying nuclei figures. We found that when the method is used for the appropriate dataset the classification performance is superior and training time is much lower compared to the other semi-supervised methods.

Our proposed method did not perform as well as TSVM when only 1 patient data was made available as the labeled set for triaging image dataset (Table 2). This is most likely due to the failure in the clustering method because an insufficient number of labeled points were made available to it. The method improved as the number of labeled points increased. Surprisingly, for the triaging image dataset, using the ssFCM method did not add any improvements to the classification performance compared to the supervised SVM method. This may be because of the fact that ssFCM assumes the underlying shape of clusters come from a Gaussian distribution; this leads to incorrect label assignment (in the cases where cluster shapes do not come from a Gaussian distribution) which in turn produces an incorrect decision boundary. Furthermore, one reason for a better performance of our method compared to the other cluster-then-label based techniques is that no assumptions are made about the underlying probability distribution of the clusters and so it can cope with clusters of any shape and form.

Although the improvements in accuracy of our proposed method compared with other techniques in Table 2 were not statistically significant after applying a Bonferonni correction, the effect size was large, with improvements of 20.4% and 25.3% in accuracy over the supervised SVM and the ssFCM methods respectively. The improvement in accuracy compared with the TSVM method was about 4% but the TSVM was very computationally expensive with each model taking more than 4 days to train.

Looking at Tables 4 and 5 for nuclei classification task, our method has a poor performance compared to the supervised SVM. The reason for this poor performance could be because the underlying structure of the data points in these datasets does not form proper clusters. This is supported by the FDR measures reported in Table 6 and t-SNE plot in Fig. 7. As shown in Table 6, the FDR measures for the relevant vs. irrelevant data has a higher value compared to L vs. BM and B vs. M data. Furthermore, from Figs 6 and 7 we can observe that relevant vs. irrelevant and L vs. BM tend to form clusters of points in the dimensionality-reduced t-SNE plots while B vs. M data does not form detectable clusters of points thus violating the cluster assumption of SSL. It is also important to note that semi-supervised learning methods are traditionally suitable for applications where only limited labeled data are available. This means that SSL methods may not work as well as supervised methods when large amounts of labeled data are present^1^.

In our preliminary experiments^30^, we systematically examined the effect of *k*, which controls the number of points that lie within the neighborhood of a labeled point, on a subset of our dataset. We found that the performance of our method was stable when a sufficiently large value was chosen for *k*. The best performance was achieved for *k* = one tenth of the number of points in the dataset.

Although TSVM is one of the top performing implementations of semi-supervised SVM, its performance was not found to be better on small-sized synthetic datasets when compared to the Branch and Bound (BB) technique^21,23^. The BB method seems to find the globally optimal solution for semi-supervised learning since it efficiently looks through all label combinations in the data space. However, due to its growing search tree basis for finding the solution, its train time is reported to be even slower than TSVM making it infeasible to apply on datasets with more than 200 data points^21^.

Our proposed semi-supervised cluster-then-label method showed improved performance over other methods for the triaging task, however, it did not perform well in the nuclei classification task. This suggests that although semi-supervised approaches may be useful in digital pathology where generating sufficiently large labeled datasets is a challenge, additional work is needed to identify whether the clustering assumptions are valid for specific tasks.
